# Expert-guided optimization for load transfer in distribution networks assisted by virtual power plants

**DOI:** 10.1371/journal.pone.0343196

**Published:** 2026-07-29

**Authors:** Lu Chen, Jinhu Fang, Xiaona Lv, Li Zhang, Yangjunran Zhou, Mingming Zhou

**Affiliations:** 1 State Grid Hefei Electric Power Supply Company, Hefei, Anhui, China; 2 Anhui University, Hefei, Anhui, China; Aalto University, FINLAND

## Abstract

The rapid expansion of distribution networks and the increasing complexity of their topological structures pose significant challenges to fast and reliable post-fault service restoration. Meanwhile, driven by carbon neutrality goals, the large-scale integration of distributed energy resources (DERs) enhances operational flexibility but also introduces pronounced intermittency and uncertainty, further complicating post-fault load transfer decision-making. To address these challenges, this paper proposes an expert-guided and virtual power plant (VPP)-assisted load transfer optimization framework based on hierarchical graph reinforcement learning. A topology-aware graph neural network (GNN)–based state representation is developed, in which buses are modeled as nodes and switches as controllable edges, enabling explicit modeling of network connectivity and electrical coupling. On this basis, a hierarchical decision-making architecture is constructed: the upper-level agent, guided by expert knowledge, dynamically selects the restoration task type to coordinate the timing of network reconfiguration and VPP-assisted DER regulation; driven by this high-level directive, two specialized lower-level agents respectively execute the specific switch operations and stepwise DER power adjustments, ensuring power balance and voltage security. Simulation results on a practical distribution network demonstrate that, under high DER penetration, the proposed method achieves faster service restoration, higher load recovery ratios, and significantly fewer voltage violation events than conventional reinforcement learning approaches, exhibiting improved operational safety and scheduling stability.

## Introduction

With the rapid development of the energy internet and the increasing level of urban electrification, large-scale distributed energy resources (DERs) have been increasingly integrated into distribution networks, driving a structural transition from traditional feeder-oriented radial systems to multi-source coupled active networks. While this transformation significantly enhances renewable energy utilization and local supply capability, it also results in more complex power flow patterns, increased operational uncertainty, and stronger coupling among network components, especially when power-electronic-interfaced systems operate under weak-grid conditions, where alternating current–direct current (AC–DC) coupling and multiport converter dynamics may affect system stability [1–3]. Consequently, distribution networks are facing growing challenges in terms of operational security, resilience, and control complexity. As distribution systems directly serve end users, outages caused by distribution-level faults account for a major proportion of power interruption events, making fast and reliable service restoration particularly critical under high DER penetration [4].

Load transfer through network reconfiguration is one of the most fundamental and effective measures for post-fault service restoration in distribution networks. By appropriately operating sectionalizing and tie switches, network topology can be rapidly restructured to maximize the recovery of de-energized areas [5]. In modern active distribution networks, controllable DER outputs can further expand the feasible supply region and improve power flow feasibility, and thus are often incorporated as auxiliary resources during restoration. However, directly controlling a large number of heterogeneous DERs at the device level during post-fault restoration can substantially increase the dimensionality and complexity of the control space, posing additional challenges for real-time and reliable decision making. Moreover, the reliable operation of power-electronic interfaces also depends on converter-level functions such as fault diagnosis and DC capacitor voltage balancing, which are essential for maintaining service continuity under abnormal operating conditions [6]. Nevertheless, with or without DER participation, load transfer inherently constitutes a mixed-integer nonlinear programming problem involving both topological reconfiguration and power dispatch [7–9]. In large-scale and highly coupled distribution networks, the resulting nonconvex feasible region and stringent real-time requirements pose significant challenges to conventional solution approaches.

Existing studies on load transfer optimization can be broadly classified into heuristic methods, mathematical optimization techniques, and expert systems [10,11]. Heuristic strategies rely on manually designed rules and offer intuitive solutions [12], yet they often lack global optimality and adaptability to complex reconfiguration scenarios. Mathematical optimization methods possess solid theoretical foundations and can explicitly model electrical constraints [13]; however, the coexistence of nonlinear power flow constraints and discrete switching operations leads to prohibitive computational burdens as network scale increases, limiting their applicability in real-time fault recovery. Expert systems enable fast responses but suffer from high knowledge engineering costs and limited flexibility in handling large-scale systems with high DER penetration [14]. These limitations indicate that traditional approaches struggle to simultaneously achieve real-time performance, accuracy, and operational feasibility in modern active distribution networks. In parallel, recent feature-driven predict-and-optimize studies have linked data-driven features with downstream network-constrained operational decisions, emphasizing decision quality rather than prediction accuracy alone [15].

In recent years, deep reinforcement learning (DRL) has emerged as a promising alternative for distribution network control due to its ability to learn decision-making policies in high-dimensional and constrained environments through direct interaction with the system [16]. Recent research has further demonstrated that graph-based reinforcement learning can explicitly exploit distribution network topology to support real-time outage management and restoration decision-making under changing operating conditions [17]. However, directly applying DRL to fault-induced load transfer still faces several critical challenges. First, load transfer decisions involve heterogeneous action spaces that combine discrete switching operations with flexible power regulation, which are difficult to handle efficiently within a single DRL architecture. Second, conventional state representations often fail to fully exploit the inherent graph structure of distribution networks, resulting in limited effectiveness when handling complex network reconfiguration states during restoration. Third, unrestricted exploration during early training stages may generate unsafe actions that violate operational constraints, such as voltage or current limits, undermining practical applicability in real power systems [18].

To reduce the complexity of power dispatch under large-scale DER integration, this study introduces a virtual power plant (VPP) aggregation model as a key enabling mechanism [19]. Unlike conventional schemes that control DERs individually, a VPP logically aggregates distributed generation units, flexible loads, and energy storage systems into a unified controllable entity with an aggregated dispatch interface [20]. Beyond local energy scheduling, recent studies have investigated the broader coordination capabilities of aggregated DERs. VPP aggregation has been employed to coordinate heterogeneous resources for inertia provision and primary frequency response [21], while multi-stage robust optimization has been developed to coordinate distribution system operators and DER aggregators participating in wholesale electricity markets under uncertainty [22]. Through this aggregation, the originally high-dimensional device-level control problem is transformed into a structured power scheduling task. Acting as an intermediate layer between the physical network and the learning agent, the VPP abstracts device-level variability and provides a consistent control interface, thereby alleviating the computational burden of high-dimensional regulation.

Building upon these insights, this paper proposes an Expert-Guided Hierarchical Graph Reinforcement Learning (EH-GRL) framework for fault recovery in active distribution networks, integrating VPP aggregation with safe policy learning. Expert knowledge is incorporated through imitation learning and rule-based operational constraints to guide early-stage exploration and ensure feasible training [23]. A graph neural network (GNN)-based state representation module is further developed to explicitly model network topology and electrical coupling [24]. Furthermore, a hierarchical control architecture is designed, where an upper-level Proximal Policy Optimization (PPO) agent provides high-level coordination, while a lower-level hybrid controller executes primitive actions: a deep Q-network (DQN) for discrete switching and a PPO-based policy for power dispatch.

The main contributions of this paper are summarized as follows:

(1) A hierarchical reinforcement learning framework is developed for service restoration in distribution networks with high penetration of distributed energy resources. By coordinating network reconfiguration and VPP-based aggregation, the proposed framework decouples discrete topological switching decisions from flexible power dispatch, thereby alleviating the complexity caused by heterogeneous and coupled action spaces.
(2) A VPP-integrated graph representation mechanism is devised to enhance state encoding efficiency under large-scale DER integration. Unlike traditional device-level mapping, aggregated resources are abstracted as controllable virtual nodes embedded within the network topology. This structural design significantly reduces representation dimensionality while preserving essential electrical coupling and topological constraints.
(3) An expert-guided collaborative learning mechanism is established by systematically integrating imitation learning with rule-based safety constraints. By incorporating expert prior knowledge to regulate early-stage exploration, the proposed mechanism ensures operational feasibility and improves the training stability and convergence efficiency of the DRL agents.

## Problem formulation

Based on the considered distribution network and post-fault restoration scenario, the service restoration problem is formulated as a Markov decision process (MDP), which provides a rigorous mathematical framework for sequential decision-making under dynamic network topology and operational uncertainty.

### Graph-based state representation

The post-fault service restoration problem is modeled as a MDP characterized by the tuple ⟨𝒮,𝒜,P,R,γ⟩, where 𝒮 denotes the state space, 𝒜 denotes the action space, $P(s_{t+1}|s_{t},a_{t})$ represents the state transition dynamics governed by the power system and restoration actions, $R(s_{t},a_{t})$ is the reward function evaluating restoration performance, and γ∈(0,1] is the discount factor. Specifically, $s_{t}\in 𝒮$ and $a_{t}\in 𝒜$ denote the system state and action at time step *t*, respectively.

Although the MDP is formulated in a general stochastic form, the transition dynamics in this study are primarily determined by the switching logic and AC power flow calculations. While the framework supports stochasticity, the simulation environment here assumes deterministic transitions unless exogenous disturbances are explicitly introduced.

To avoid ambiguity between graph learning and physical simulation, two graph representations are defined at each time step: a physics graph for network simulation and constraint enforcement, and a learning graph for graph neural network inference.

The physics graph is defined as


$$\begin{array}{c} {𝒢}_{t}^{phy}=({𝒱}_{bus},{ℰ}_{t}^{phy},{𝐗}_{t}^{phy},{𝐄}_{attr}^{phy}), \end{array}$$ (1)

where ${𝒱}_{bus}$ denotes the set of physical buses, ${ℰ}_{t}^{phy}$ denotes the set of energized physical branches and switches at time step *t*, ${𝐗}_{t}^{phy}$ denotes the physical node-state feature matrix, and ${𝐄}_{attr}^{phy}$ denotes *t*he physical edge-attribute matrix containing branch parameters such as line resistance and reactance. This graph is exclusively used for AC power flow calculation, radiality checking, and operational constraint enforcement.

To facilitate information aggregation between DERs and the grid in the graph attention network (GAT), a learning graph is further constructed as


$$\begin{array}{c} {𝒢}_{t}^{learn}=(𝒱,{ℰ}_{t}^{learn},{𝐗}_{t},{𝐄}_{attr}^{learn}), \end{array}$$ (2)

where


$$\begin{array}{c} 𝒱={𝒱}_{bus}\cup {𝒱}_{DER}, \end{array}$$ (3)

and


$$\begin{array}{c} {ℰ}_{t}^{learn}={ℰ}_{t}^{phy}\cup {ℰ}^{virt}. \end{array}$$ (4)

Here, ${𝒱}_{DER}$ denotes the set of virtual DER nodes, and ${ℰ}^{virt}$ denotes the set of virtual connection edges linking each DER virtual node to its point of common coupling (PCC).

It should be emphasized that the virtual edges in ${ℰ}^{virt}$ are introduced solely for message passing and state representation learning in the GAT. They are not physical branches and are therefore excluded from AC power flow equations, topology feasibility checks, radiality constraints, and branch flow limit evaluation. In particular, the learning graph inherits all physical buses and physical branches from the physics graph, and further augments them with DER virtual nodes and virtual edges. In this way, the two graphs remain consistent through their shared physical subgraph while serving different purposes in neural inference and physical simulation.

Each node i∈𝒱 is associated with a feature vector ${𝐱}_{i}$, defined as


$$\begin{array}{c} {𝐱}_{i}=\left\{ \begin{array}{cc} {\left[V_{i},\;P_{i}^{load},\;{𝕀}_{i}^{src}\right]}^{T}, & i\in {𝒱}_{bus},\\ {𝐬}_{i}^{DER}, & i\in {𝒱}_{DER}. \end{array}\right. \end{array}$$ (5)

where ${𝐬}_{i}^{DER}$ includes device-specific operational states such as available power capacity, state-of-charge, and controllability indicators. Consequently, the global node feature matrix is constructed as ${𝐗}_{t}\in {\mathbb{R}}^{|𝒱|\times d_{v}}$.

Each physical branch $(i,j)\in {ℰ}_{t}^{phy}$ is associated with an attribute vector


$$\begin{array}{c} {𝐞}_{ij}=[\;r_{ij},\;x_{ij}\;{]}^{T}. \end{array}$$ (6)

For physical branches, $r_{ij}$ and $x_{ij}$ denote the actual line resistance and reactance, respectively. For virtual edges in ${ℰ}^{virt}$, the corresponding edge attributes are assigned as a negligible constant ϵ≈0 only as placeholder edge attributes for neural message passing, rather than as physical impedance parameters in the power flow model.

The collection of physical branch attribute vectors forms the physical edge-attribute matrix


$$\begin{array}{c} {𝐄}_{attr}^{phy}\in {\mathbb{R}}^{|{ℰ}_{t}^{phy}|\times 2}, \end{array}$$ (7)

while the collection of all edge attribute vectors in the learning graph forms


$$\begin{array}{c} {𝐄}_{attr}^{learn}\in {\mathbb{R}}^{|{ℰ}_{t}^{learn}|\times 2}. \end{array}$$ (8)

Here, each row corresponds to the transposed edge attribute vector ${𝐞}_{ij}^{T}$ associated with an edge in the corresponding graph.

Accordingly, the state input to the policy network is represented by the learning graph


$$\begin{array}{c} s_{t}≜{𝒢}_{t}^{learn}, \end{array}$$ (9)

while AC power flow calculation, environment transition, and feasibility verification are carried out on the physics graph ${𝒢}_{t}^{phy}$.

During each restoration step, the selected switching action is first applied to ${𝒢}_{t}^{phy}$ by updating the energized physical branch set ${ℰ}_{t}^{phy}$. Topology masks, radiality constraints, and operational feasibility checks are then evaluated only on the updated physics graph. After the AC power flow is solved and the new physical operating state is obtained, the learning graph for the next decision step, ${𝒢}_{t+1}^{learn}$, is reconstructed by augmenting the updated physics graph ${𝒢}_{t+1}^{phy}$ with DER virtual nodes and virtual edges.

### Hybrid action space and state transition

To enable coordinated control of network reconfiguration and resource scheduling, the action space is constructed as a heterogeneous action space defined as


$$\begin{array}{c} 𝒜={𝒜}_{switch}\cup {𝒜}_{DER}. \end{array}$$ (10)

At each decision step, the hierarchical agent selects a single action $a_{t}\in 𝒜$. If $a_{t}\in {𝒜}_{switch}$, it represents a switching operation that modifies the network topology ${ℰ}_{t}$. If $a_{t}\in {𝒜}_{DER}$, it corresponds to an active power regulation command for the virtual power plant (VPP).

The VPP is modeled as an aggregated entity. This aggregation provides a tractable abstraction for system-level decision-making, while preserving essential physical constraints through time-varying power boundaries.

Its active power injection $P_{VPP,i}(t)$ is constrained by the aggregated physical limits of its internal components:


$$\begin{array}{cc} P_{VPP,i}^{\max}(t) & =\sum_{k\in \Omega_{PV}}P_{PV,k}^{\max}(t)+\sum_{j\in \Omega_{ESS}}P_{dis,j}^{\max}(t)-\sum_{m\in \Omega_{EV}}P_{EV,m}^{\min}(t), \end{array}$$ (11a)

$$\begin{array}{cc} P_{VPP,i}^{\min}(t) & =0-\sum_{j\in \Omega_{ESS}}P_{ch,j}^{\max}(t)-\sum_{m\in \Omega_{EV}}P_{EV,m}^{\max}(t). \end{array}$$ (11b)

Here, $P_{PV,k}^{\max}(t)$ denotes the maximum available photovoltaic power determined by solar irradiance. The zero term in the lower bound explicitly indicates that PV output is fully curtailable if required. The ESS limits are constrained by inverter ratings and state-of-charge. For EVs, the charging flexibility is constrained by the user’s energy demand and departure time.

Specifically, the immediate charging boundaries ($P_{EV,m}^{\min},P_{EV,m}^{\max}$) are dynamically updated to ensure that the required energy $E_{req,m}$ is satisfied before departure, thereby enforcing the energy sufficiency constraint through the aggregated boundary model.

For VPP regulation, the DER action set is defined as


$$\begin{array}{c} {𝒜}_{DER}=\{-\delta ,0,+\delta \}, \end{array}$$ (12)

where δ denotes a predefined normalized adjustment ratio.

At each time step *t*, the selected action $a_{t}^{DER}\in {𝒜}_{DER}$ represents a relative decrease, hold, or increase command with respect to the adjustable capacity of the VPP.

The actual power setpoint of VPP *i* is updated as


$$\begin{array}{c} P_{VPP,i}(t+1)={\mathrm{Proj}}_{\left[P_{VPP,i}^{\min},\;P_{VPP,i}^{\max}\right]}\left(P_{VPP,i}(t)+a_{t}^{DER}\cdot P_{VPP,i}^{cap}\right), \end{array}$$ (13)

where $P_{VPP,i}^{cap}$ denotes the available adjustable capacity of VPP *i*, and $P_{VPP,i}^{\min}$ and $P_{VPP,i}^{\max}$ are the minimum and maximum admissible power limits, respectively.

The projection operator enforces feasibility by ensuring that the updated power setpoint remains within the VPP operational bounds.

The VPP-level action generated by the VPP-control agent serves as an aggregate regulation command for the internal DER resources. After the aggregate active-power setpoint of VPP *i* is updated, the requested VPP-level power adjustment is calculated as


$$\begin{array}{c} \Delta P_{VPP,i}^{req}(t)=P_{VPP,i}(t+1)-P_{VPP,i}(t), \end{array}$$ (14)

where $P_{VPP,i}(t+1)$ is the updated aggregate active-power setpoint obtained from the VPP-level regulation command, $P_{VPP,i}(t)$ is the current active-power injection of VPP *i*, and $\Delta P_{VPP,i}^{req}(t)$ denotes the requested aggregate power adjustment to be realized by the internal DER resources.

To physically realize this VPP-level regulation command, the controllable DER units inside VPP *i* are collected as


$$\begin{array}{c} {𝒟}_{i}=\{{PV}_{1},\ldots ,{PV}_{N_{PV,i}},{ESS}_{1},\ldots ,{ESS}_{N_{ESS,i}},{EV}_{1},\ldots ,{EV}_{N_{EV,i}}\}, \end{array}$$ (15)

where ${𝒟}_{i}$ denotes the set of controllable DER units in VPP *i*, including PV units, ESS units, and EV charging units. Here, $N_{PV,i}$, $N_{ESS,i}$, and $N_{EV,i}$ denote the numbers of controllable PV units, ESS units, and EV charging units contained in VPP *i*, respectively.

The requested aggregate adjustment is realized by updating the internal DER setpoints subject to their real-time regulation margins and operating constraints. The realized aggregate VPP power adjustment after device-level allocation can be expressed as


$$\begin{array}{c} \Delta P_{VPP,i}^{rea}(t)=\sum_{k\in \Omega_{PV}}\Delta P_{k}^{PV}(t)+\sum_{j\in \Omega_{ESS}}\Delta P_{j}^{ESS}(t)-\sum_{m\in \Omega_{EV}}\Delta P_{m}^{EV}(t), \end{array}$$ (16)

where $\Delta P_{VPP,i}^{rea}(t)$ is the realized aggregate VPP power adjustment after intra-VPP allocation, and $\Delta P_{k}^{PV}(t)$, $\Delta P_{j}^{ESS}(t)$, and $\Delta P_{m}^{EV}(t)$ denote the setpoint adjustments of PV, ESS, and EV units, respectively. The negative sign before the EV term is used because EV charging is modeled as controllable load. Therefore, reducing EV charging demand increases the net VPP active-power injection, whereas increasing EV charging demand decreases it.

After the VPP-level regulation command is determined by the VPP-control agent, it is passed to the intra-VPP dispatch module for physical implementation. According to the real-time operating constraints, the intra-VPP dispatch module first evaluates the feasible regulation margins of controllable PV, ESS, and EV units. Then, the module identifies the DER units that can contribute to the required regulation direction and determines their allocation weights according to their available regulation margins and predefined dispatch priority coefficients. Based on the normalized allocation weights, the aggregate VPP-level regulation signal is allocated to one or more feasible units. Each device-level regulation command is bounded by the corresponding device operating range during execution. When the regulation margin of some units is insufficient, the dispatch module further selects other units with feasible margins to share the remaining adjustment, thereby improving the executability of the aggregate command. Finally, the obtained device-level commands are used to update the setpoints of PV active-power output, ESS charging/discharging power, and EV charging load.

When $a_{t}^{DER}>0$, the VPP is required to increase its net active-power injection. The admissible device-level realizations include increasing PV output when curtailed power is available, increasing ESS discharging power or reducing ESS charging power, and reducing EV charging demand. Conversely, when $a_{t}^{DER}<0$, the VPP is required to decrease its net active-power injection. The admissible device-level realizations include curtailing PV output, reducing ESS discharging power or increasing ESS charging power, and increasing EV charging demand. When $a_{t}^{DER}=0$, the current DER setpoints are maintained.

After the device-level realization is determined by the intra-VPP dispatch module, the corresponding DER setpoints are adjusted in the same normalized incremental manner as the VPP-level command and then projected onto their admissible operating ranges. For PV units, the admissible range is determined by the available photovoltaic power and curtailment limits. For ESS units, the admissible range is determined by the charging/discharging power ratings and SOC constraints. For EV units, the admissible range is determined by the charging power bounds and the remaining energy requirement before departure. After the bounded device-level updates, the realized active-power injection of VPP *i* is recalculated by aggregating the updated internal DER setpoints. Therefore, although the learned policy outputs the VPP-level aggregate regulation command, the physical execution is explicitly mapped to device-level DER operations, including the adjusted DER units, their regulation directions, and their setpoint updates.

The system state transition is implemented according to the selected action. If a switching action is selected, the physical topology ${ℰ}_{t}^{phy}$ is updated. If a DER regulation action is selected, the internal DER setpoints are updated through the intra-VPP dispatch module according to the corresponding device-level realization rule. Then, the environment solves the AC power flow on the updated physics graph ${𝒢}_{t+1}^{phy}$ to obtain the new operating point, including nodal voltages and branch power flows. Finally, the learning graph ${𝒢}_{t+1}^{learn}$ is constructed by augmenting the updated physics graph with DER virtual nodes and virtual edges for state encoding.

Therefore, the physical transition is written as


$$\begin{array}{c} {𝒢}_{t+1}^{phy}=f_{phy}({𝒢}_{t}^{phy},a_{t}), \end{array}$$ (17)

where $f_{phy}(\cdot )$ denotes the environment-level physical transition mapping, which updates the physical network topology or the VPP aggregate power injection according to the selected action $a_{t}$, and then solves the AC power flow to obtain the updated physical operating point. The state used by the agent at the next step is constructed as


$$\begin{array}{c} s_{t+1}≜{𝒢}_{t+1}^{learn}=f_{learn}({𝒢}_{t+1}^{phy},{𝐬}_{t+1}^{DER}), \end{array}$$ (18)

where $f_{learn}(\cdot )$ denotes the learning-graph construction mapping, which augments the updated physical graph with DER virtual nodes, virtual edges, and the updated DER operating states for state encoding.

### Operational constraints and multi-objective reward design

All operational constraints are imposed on the physics graph ${𝒢}_{t}^{phy}$. In particular, the branch-flow equations, voltage magnitude limits, branch thermal limits, and radiality requirement are defined only over physical buses and physical branches in ${ℰ}_{t}^{phy}$. Virtual edges in the learning graph are excluded from all physical feasibility checks.

Given a control action, the simulation environment evaluates the post-action operating state on the physics graph through AC power flow analysis. For radial distribution systems, the corresponding network constraints can be expressed in the following branch-flow form.

At each time step *t*, for each physical branch $(i,j)\in {ℰ}_{t}^{phy}$, the network variables satisfy the following power balance and voltage drop equations:


$$\begin{array}{cc} P_{ij,t} & =\sum_{k\in {𝒩}_{j}}P_{jk,t}+\alpha_{j,t}P_{j,t}^{load}-P_{j,t}^{DER}+r_{ij}\frac{P_{ij,t}^{2}+Q_{ij,t}^{2}}{V_{i,t}^{2}}, \end{array}$$ (19)

$$\begin{array}{cc} Q_{ij,t} & =\sum_{k\in {𝒩}_{j}}Q_{jk,t}+\alpha_{j,t}Q_{j,t}^{load}-Q_{j,t}^{DER}+x_{ij}\frac{P_{ij,t}^{2}+Q_{ij,t}^{2}}{V_{i,t}^{2}}, \end{array}$$ (20)

$$\begin{array}{cc} V_{j,t}^{2} & =V_{i,t}^{2}-2(r_{ij}P_{ij,t}+x_{ij}Q_{ij,t})+(r_{ij}^{2}+x_{ij}^{2})\frac{P_{ij,t}^{2}+Q_{ij,t}^{2}}{V_{i,t}^{2}}, \end{array}$$ (21)

The last terms in Eqs. (19)–(21) represent the quadratic power losses and voltage drops on branch (*i*,*j*), with resistance $r_{ij}$ and reactance $x_{ij}$.

Given a control action, the simulation environment solves the full AC power flow equations (e.g., via the Newton–Raphson method) to obtain the system operating point, which is then used to verify operational security.

Specifically, nodal voltage magnitudes must remain within permissible bounds,


$$\begin{array}{c} V_{\min}\leq V_{i,t}\leq V_{\max}, \end{array}$$ (22)

and branch apparent power flows must respect thermal ratings,


$$\begin{array}{c} \sqrt{P_{ij,t}^{2}+Q_{ij,t}^{2}}\leq S_{ij}^{\max}. \end{array}$$ (23)

Regarding resource capability, the dispatch of DERs is constrained by their inverter capacity $S_{i}^{DER}$ and active power limits $\left[P_{i}^{DER,min},P_{i}^{DER,max}\right]$, defined as:


$$\begin{array}{c} \sqrt{(P_{i,t}^{DER}{)}^{2}+(Q_{i,t}^{DER}{)}^{2}}\leq S_{i}^{DER},\;P_{i}^{DER,min}\leq P_{i,t}^{DER}\leq P_{i}^{DER,max}. \end{array}$$ (24)

Additionally, the network topology is strictly constrained to maintain a radial structure for coordination with protection schemes.

Within the proposed framework, hard constraints, including radiality and switching feasibility, are enforced via action masking over controllable physical switches only. Virtual edges are fixed auxiliary connections in the learning graph and are not subject to switching actions or topology constraints. Soft constraints (e.g., voltage violations and thermal overloads) are strictly penalized in the reward function.

The reward function is defined as


$$\begin{array}{c} R_{t}=\alpha \cdot r_{rec}({𝒢}_{t})-\beta \cdot r_{loss}({𝒢}_{t})+\lambda_{safe}\cdot r_{safe}({𝒢}_{t})-\mu \cdot r_{cost}(a_{t}^{DER}), \end{array}$$ (25)

where $r_{rec}$ represents the proportion of restored load, $r_{loss}$ penalizes active power losses, and $r_{cost}$ accounts for DER operational and degradation costs. The specific values of the reward-related hyperparameters used in the experiments are summarized in Table 1. These coefficients were selected based on preliminary trials and the relative scales of different reward components, so as to emphasize load restoration and physically feasible operating states while retaining line-loss reduction and DER regulation cost as auxiliary objectives.

**Table 1:** Experimental hyperparameters used in this study.

Hyperparameter	Value	Hyperparameter	Value
Total training episodes	5000	DQN batch size	32
Maximum decision steps per episode	10	DQN learning rate	0.001
Reward coefficient α	2	DQN discount factor γ	0.95
Reward coefficient β	0.5	Initial epsilon	0
Reward coefficient $\lambda_{safe}$	5.0	Maximum epsilon	0.99
Reward coefficient μ	0.5	Epsilon increment	0.001
Minimum voltage limit	0.95	Replay memory capacity	3000
Maximum voltage limit	1.05	Soft target update coefficient τ	0.005
		DQN gradient clipping	1.0
PPO learning rate	$1\times 10^{-4}$	Behavior cloning epochs	20
PPO discount factor γ	0.99	Behavior cloning batch size	32
PPO epochs	4	Behavior cloning learning rate	$7\times 10^{-4}$
PPO mini-batch size	32	Validation ratio	0.2
PPO clip parameter	0.2	Imitation coefficient	0.5
Value loss coefficient	0.5		
PPO entropy coefficient	0.01		
PPO gradient clipping	0.5		

The safety-related discrete reward component $r_{safe}({𝒢}_{t})$ is defined as:


$$\begin{array}{c} r_{safe}({𝒢}_{t})=\left\{ \begin{array}{cc} +1, & \text{if all restorable loads are energized and all operational constraints are satisfied},\\ -1, & \text{if operational constraints (e.g., voltage) are violated},\\ 0, & \text{otherwise}. \end{array}\right. \end{array}$$ (26)

Here, $\lambda_{safe}>0$ is a weighting coefficient that scales this explicit feedback to guide the agent toward physically feasible operating states.

The optimal control policy is obtained by maximizing the expected cumulative discounted return:


$$\begin{array}{c} \pi_{\theta }^{*}=\text{arg}\max_{\theta }\;{𝔼}_{\pi_{\theta }}\left[\sum_{t=0}^{T}\gamma^{t}R_{t}\right], \end{array}$$ (27)

which formalizes the objective of identifying a coordinated sequence of topology reconfiguration and DER dispatch actions to achieve rapid, efficient, and secure post-fault restoration.

## Proposed hierarchical graph reinforcement learning framework

Building upon the graph-based MDP formulation presented in the previous section, this section presents the proposed Expert-Guided Hierarchical Graph Reinforcement Learning (EH-GRL) framework for post-fault restoration in active distribution networks.

**Fig 1:**
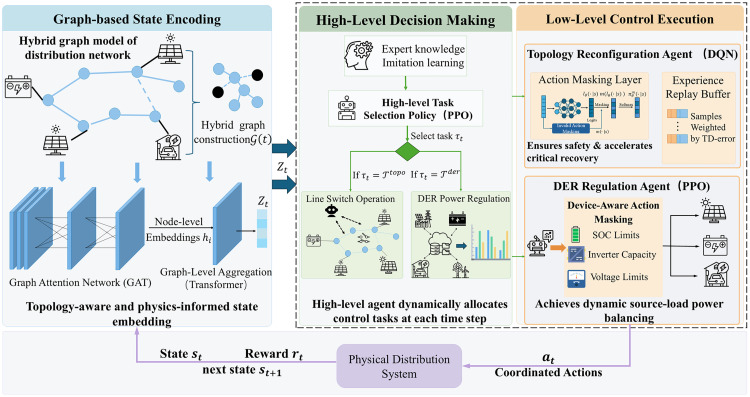
Architecture of the proposed Expert-Guided Hierarchical Graph Reinforcement Learning (EH-GRL) framework. A hybrid state encoder—integrating a Graph Attention Network with Transformer-based aggregation—extracts topology-aware features from the graph state ${𝒢}_{t}$. These features are fed into an expert-guided high-level PPO agent, trained with imitation learning from expert demonstrations, to select the control task. The selected task activates specialized low-level agents—DQN for topology reconfiguration and PPO for DER dispatch—which execute feasible actions while employing physics-informed action masking to enforce operational constraints.

As illustrated in Fig 1, the proposed EH-GRL framework adopts a hierarchical architecture integrating a hybrid graph-based state encoder, expert-guided high-level task selection, and specialized low-level controllers for safe and efficient restoration.

### Hierarchical reinforcement learning framework for coordinated restoration

This subsection describes the internal decision-making process of the proposed Expert-Guided Hierarchical Graph Reinforcement Learning (EH-GRL) framework. The post-fault restoration problem involves heterogeneous and tightly coupled control decisions, including discrete network topology reconfiguration and stepwise incremental DER power regulation. Directly optimizing a composite action space that simultaneously covers both decision types leads to a combinatorial explosion and unstable policy learning. To address this challenge, EH-GRL adopts a hierarchical decision-making architecture that separates strategic task allocation from safety-aware execution, thereby reducing decision complexity while preserving coordination between structural and operational restoration actions [25].

At each decision step, the environment provides the current graph state ${𝒢}_{t}$, which is encoded into the latent representation ${𝐳}_{t}$ by the shared GAT–Transformer encoder. Based on ${𝐳}_{t}$, the high-level policy selects a task type $\tau_{t}$, determining whether the control focus should be placed on topology reconfiguration or DER regulation. Conditioned on the selected task, the corresponding low-level controller generates a feasible action within its task-specific action subspace. Actions associated with the inactive task are masked out by assigning −∞ logits (or Q-values), ensuring that only task-consistent decisions are executed. The selected action is then applied to the environment, leading to a state transition and a scalar reward reflecting restoration progress, operational safety, and constraint satisfaction. This shared reward signal is used to update both the high-level task-selection policy and the active low-level controller, thereby enabling long-term priority allocation and safe task-specific execution across hierarchical levels.

The high-level policy operates on a compact graph-level state representation, denoted as ${𝐳}_{t}$, which is extracted by a topology-aware GAT–Transformer encoder. Specifically, we denote the graph encoding function implemented by the GAT–Transformer encoder as $f_{enc}(\cdot )$. In accordance with the state modeling introduced in the previous section, the environment state at time step *t* is represented as a graph ${𝒢}_{t}$. All decision-making policies in the proposed framework operate on the encoded representation


$$\begin{array}{c} {𝐳}_{t}=f_{enc}({𝒢}_{t}), \end{array}$$ (28)

rather than directly on the raw graph structure.

Instead of directly generating the composite action $a_{t}$, the high-level policy first selects a restoration task type:


$$\begin{array}{c} \tau_{t}\in \{{𝒯}^{topo},\;{𝒯}^{DER}\}, \end{array}$$ (29)

where $\tau_{t}$ is the selected task type at time step *t*. ${𝒯}^{topo}$ represents a task focused on topology reconfigura*t*ion, which involves structural changes to the network, while ${𝒯}^{DER}$ corresponds to a task focused on DER regulation, optimizing the operation of energy resources within the network. This task-level gating mechanism dynamically allocates control focus between topology reconfiguration and DER regulation based on the evolving network condition. By doing so, it effectively decouples the discrete structural decisions (topology changes) from operational power support actions (DER regulation), all while maintaining a unified MDP formulation.

Accordingly, only the sub-action associated with the selected task is activated at each step. When $\tau_{t}={𝒯}^{topo}$, a low-level topology controller generates the switching action $a_{t}^{switch}$; when $\tau_{t}={𝒯}^{DER}$, a DER controller produces the regulation action $a_{t}^{DER}$, while the other sub-action remains inactive. This hierarchical execution scheme preserves the original environment action interface but substantially reduces the effective action space explored at each step, improving learning efficiency and stability during long restoration horizons.

Importantly, although the decision logic is hierarchically structured, the underlying environment exposes a unified discrete action space. Specifically, the global action space 𝒜 comprises mutually disjoint subsets corresponding to network reconfiguration and DER regulation, and since these subsets are mutually exclusive, each action uniquely determines its associated task category. Consequently, the hierarchical decomposition is realized internally through task-conditioned action masking, rather than by modifying the fundamental interaction interface with the environment.

The high-level policy is parameterized as a categorical distribution


$$\begin{array}{c} \pi_{\theta }(\tau_{t}|{𝐳}_{t})=softmax({𝐖}_{high}^{T}{𝐳}_{t}+{𝐛}_{high}), \end{array}$$ (30)

where $\tau_{t}$ denotes the high-level task selected at time step *t*, ${𝐳}_{t}$ is the global graph-level state embedding ex*t*racted by the encoder, ${𝐖}_{high}$ and ${𝐛}_{high}$ denote the weight matrix and bias vector of the linear output layer, respectively, and $\theta =\{{𝐖}_{high},{𝐛}_{high}\}$ represents the learnable parameters of the high-level policy.

To improve early-stage convergence and reduce exploration variance, the high-level policy is initialized via the proposed expert-guided imitation learning strategy, and subsequently optimized using the Proximal Policy Optimization (PPO) objective.


$$\begin{array}{c} J_{PPO}(\theta )={𝔼}_{t}[\min(r_{t}(\theta ){\hat{A}}_{t},clip(r_{t}(\theta ),1-\epsilon ,1+\epsilon ){\hat{A}}_{t})], \end{array}$$ (31)

where ${𝔼}_{t}[\cdot ]$ denotes the expectation over sampled time steps, ϵ is the PPO clipping threshold, $r_{t}(\theta )=\frac{\pi_{\theta }(\tau_{t}|{𝐳}_{t})}{\pi_{\theta_{old}}(\tau_{t}|{𝐳}_{t})}$ is the probability ratio between the current policy and the previous policy with parameters $\theta_{old}$, and ${\hat{A}}_{t}$ is the estimated advantage function.

In both the high-level task-selection policy and the low-level DER controller, the advantage function ${\hat{A}}_{t}$ is estimated using Generalized Advantage Estimation (GAE) with the corresponding independent critic network.

Once the task type is determined, execution is delegated to specialized low-level controllers that explicitly enforce physical feasibility. When topology reconfiguration is selected, a DQN-based controller explores the discrete and combinatorial switching action space. A physics-informed action masking mechanism is applied to exclude infeasible operations. Specifically, at each time step, the environment computes the feasible action set based on the current graph state ${𝒢}_{t}$, excluding actions on faulted components, violations of network radiality, and switching sequences that may induce electrical islands.

The masked Q-values are defined as


$$\begin{array}{c} Q_{masked}({𝐳}_{t},a)=\left\{ \begin{array}{cc} Q({𝐳}_{t},a), & \text{if }a\in {𝒜}_{feasible}({𝒢}_{t}),\\ -\infty , & \text{otherwise}, \end{array}\right. \end{array}$$ (32)

where *a* is a candidate switching action, $Q({𝐳}_{t},a)$ is the action-value function, and ${𝒜}_{feasible}({𝒢}_{t})$ represents the set of physically feasible actions determined by the environment under the current network configuration.

These masked Q-values are consistently used during both action selection and target value computation. The topology controller is trained by minimizing the temporal-difference loss


$$\begin{array}{c} {ℒ}_{DQN}={𝔼}_{\left({𝐳}_{t},a,r,{𝐳}_{t+1}\right)}[(r+\gamma \max_{a^{\prime }\in {𝒜}_{feasible}({𝒢}_{t+1})}Q_{target}({𝐳}_{t+1},a^{\prime })-Q({𝐳}_{t},a){)}^{2}], \end{array}$$ (33)

where *r* is the immediate reward, ${𝐳}_{t+1}$ denotes the encoded next state, γ∈(0,1) is the discount factor, and $Q_{target}(\cdot )$ is the target Q-network used to stabilize training.

When DER regulation is selected, a PPO-based controller performs stepwise power adjustments of heterogeneous DER units. Instead of directly selecting absolute power setpoints, the DER action $a_{t}^{DER}$ represents discrete incremental changes in active power, facilitating stable learning and smooth regulation under uncertainty.

PPO is applied here to a discrete categorical policy over incremental DER adjustment actions. The DER policy is parameterized as


$$\begin{array}{c} a_{t}^{DER}\sim \pi_{\phi }(a|{𝐳}_{t}), \end{array}$$ (34)

where ϕ represents the policy parameters.

The DER controller is optimized using the PPO objective


$$\begin{array}{c} J_{DER}(\phi )={𝔼}_{t}[\min(r_{t}(\phi ){\hat{A}}_{t},clip(r_{t}(\phi ),1-\epsilon ,1+\epsilon ){\hat{A}}_{t})], \end{array}$$ (35)

where $r_{t}(\phi )=\frac{\pi_{\phi }(a_{t}^{DER}|{𝐳}_{t})}{\pi_{\phi_{old}}(a_{t}^{DER}|{𝐳}_{t})}$ is the policy probability ratio and $\phi_{old}$ denotes the parameters of the previous DER policy.

The high-level task-selection policy and the low-level DER controller are equipped with independent critic networks for advantage estimation, avoiding value function interference across different decision timescales and action semantics.

A device-aware action masking mechanism is applied directly to the policy logits, in which infeasible actions are assigned −∞ log-probabilities based on the heterogeneous physical constraints of different DER types. Similar to topology reconfiguration, the corresponding feasibility masks are generated by the environment based on the current graph state ${𝒢}_{t}$. For energy storage systems and electric vehicles, the masking rules enforce state-of-charge bounds, charging and discharging power limits, and mutually exclusive operating modes. For photovoltaic units, actions exceeding the instantaneous available generation determined by irradiance conditions and inverter capacity are excluded. Through this capability-aware masking scheme, the DER controller is restricted to a dynamically feasible action set, ensuring strict compliance with device-level operational constraints.

In addition, system-level operational constraints, including voltage magnitude limits and branch thermal ratings, are enforced through a reward penalty mechanism, guiding the agent toward safe operating regions without requiring explicit constraint checks during action selection.

Overall, the proposed EH-GRL framework achieves an effective separation between strategic task allocation and safety-aware execution. The high-level policy adaptively allocates control priority between topology reconfiguration and DER regulation, while the specialized low-level controllers ensure that all executed actions remain feasible and physically compliant throughout the restoration process.

### Topology-aware state embedding via GAT

As a core perception module of the proposed EH-GRL framework, a topology-aware state embedding is constructed to encode the structural and operational characteristics of post-fault load transfer in active distribution networks. Unlike conventional grid modeling, the state representation here must account for the heterogeneous integration of VPPs. Based on the graph-based MDP formulation introduced earlier, the environment state at time step *t* is represented as a graph ${𝒢}_{t}$. Each node feature vector explicitly encodes both its functional role (e.g., load bus, DER, switch) and its real-time operational state, including voltage magnitude, load supply status, and critical VPP constraints (e.g., State of Charge, inverter capacity margin). In this dynamic graph, load transfer feasibility is jointly constrained by local electrical states and global network connectivity. These irregular, topology-dependent, and resource-constrained dependencies render conventional convolution-based architectures unsuitable.

To effectively capture such interactions, a GAT is adopted as the backbone of the state encoder. The core motivation for employing GAT in this specific context lies in its ability to explicitly model the heterogeneous electrical influence between components. In a post-fault scenario, controllable elements such as VPPs and tie switches typically exert a stronger impact on restoration feasibility than passive load buses. The attention mechanism naturally mimics this physical logic by adaptively weighting neighboring information based on electrical proximity and operational relevance (e.g., highlighting paths with sufficient VPP headroom).

Specifically, let ${𝐡}_{i}^{\left(l\right)}$ denote the embedding of node *i* at layer *l*, where ${𝐡}_{i}^{\left(0\right)}={𝐱}_{i}$ denotes the initial node feature vector. The unnorma*l*ized attention coefficient between node *i* and its neighbor *j* is computed as:


$$\begin{array}{c} e_{ij}^{\left(l\right)}=\mathrm{LeakyReLU}\left({𝐚}^{(l)⊤}[{𝐖}^{\left(l\right)}{𝐡}_{i}^{\left(l\right)}\;\|\;{𝐖}^{\left(l\right)}{𝐡}_{j}^{\left(l\right)}]\right), \end{array}$$ (36)

where ${𝐖}^{\left(l\right)}$ and ${𝐚}^{\left(l\right)}$ are trainable parameters. Physically, $e_{ij}^{\left(l\right)}$ reflects the relative relevance of power flow capability or information exchange from component *j* to *i*. These coefficients are normalized to obtain attention weights:


$$\begin{array}{c} \alpha_{ij}^{\left(l\right)}=\frac{\exp\left(e_{ij}^{\left(l\right)}\right)}{\sum_{k\in 𝒩(i)\cup \{i\}}\exp\left(e_{ik}^{\left(l\right)}\right)}, \end{array}$$ (37)

where 𝒩(i) denotes the set of neighboring nodes, and the self-loop term allows each node to retain its own operational state during aggregation. Through learning, $\alpha_{ij}^{\left(l\right)}$ evolves to highlight valid energization paths, effectively filtering out infeasible connections in the faulted network.

To enhance representational capacity, a multi-head attention mechanism with *K* heads is employed. The updated node embedding is obtained by concatenating the outputs of all attention heads:


$$\begin{array}{c} {𝐡}_{i}^{\left(l+1\right)}=\mathrm{LeakyReLU}\left(\overset{K}{\underset{k=1}{\|}}\sum_{j\in 𝒩(i)\cup \{i\}}\alpha_{ij}^{\left(l,k\right)}{𝐖}^{\left(l,k\right)}{𝐡}_{j}^{\left(l\right)}\right), \end{array}$$ (38)

where $\alpha_{ij}^{\left(l,k\right)}$ and ${𝐖}^{\left(l,k\right)}$ correspond to the attention weight and linear transformation of the *k*-th attention head, respectively.

A critical challenge in VPP-assisted restoration is preserving the boundary constraints of flexible resources. Deep GNN layers tend to over-smooth node features, which poses a risk of blurring the precise status of VPPs (e.g., distinguishing a fully charged ESS from a depleted one). To mitigate this, a residual connection from the original node features is introduced:


$$\begin{array}{c} {\tilde{𝐡}}_{i}={𝐡}_{i}^{\left(L\right)}+{𝐖}_{res}{𝐱}_{i}, \end{array}$$ (39)

where ${𝐱}_{i}$ denotes the initial feature vector of node *i*, and ${𝐖}_{res}$ is a learnable linear projection for dimension alignment. This residual design ensures that critical low-level electrical attributes are preserved for accurately evaluating load transfer feasibility under safety constraints.

While the GAT encoder captures local electrical couplings, switching operations often induce global voltage profile changes through long-range dependencies. For example, closing a tie switch in an upstream feeder may indirectly enable downstream load pickup through a sequence of power flow redistributions. To explicitly model such non-local and multi-step couplings, the node embeddings generated by the GAT are further integrated with a Transformer-based encoder within the overall EH-GRL architecture, enabling global information aggregation across the network.

Finally, the node-level representations are aggregated via a global pooling operation to obtain a compact graph-level embedding ${𝐳}_{t}=f_{enc}({𝒢}_{t})$. This global vector encapsulates both the topological connectivity and the aggregate flexibility of VPPs, providing a comprehensive context for the high-level agent to arbitrate between topological reconfiguration and DER dispatch tasks.

### Expert-guided training strategy

To facilitate efficient and stable training of the proposed EH-GRL framework, an expert-guided imitation learning (IL) strategy is introduced to initialize the high-level task-selection policy [26]. In post-fault restoration tasks, purely reward-driven reinforcement learning often suffers from low sample efficiency and unstable early-stage behavior due to the high dimensionality of system states and strict operational constraints. To address these challenges, expert demonstrations derived from restoration trajectory logs generated in the OpenDSS-based simulation environment are leveraged to provide a reliable warm start for policy learning. By distilling feasible and high-quality expert decisions into the neural policy, this strategy effectively guides early exploration toward safe and meaningful regions of the action space, thereby accelerating convergence without replacing the subsequent reinforcement learning phase.

Consistent with the unified action interface, expert demonstrations are provided at the level of concrete control actions rather than abstract task labels. Each expert action $a_{i}^{exp}$ belongs to exactly one such subset, thereby implicitly determining the corresponding task type.

Formally, the expert dataset is defined as


$$\begin{array}{c} {𝒟}_{exp}=\{({𝒢}_{i},a_{i}^{exp}){\}}_{i=1}^{N}, \end{array}$$ (40)

where ${𝒢}_{i}$ denotes the graph-structured system state, and $a_{i}^{exp}$ represents the expert-selected control action.

The expert demonstration dataset ${𝒟}_{exp}$ is constructed from restoration trajectory logs generated in the OpenDSS-based distribution system simulation environment. In the current dataset version, 1000 candidate trajectories are first generated, from which 180 trajectories are retained after quality scoring and balanced screening. The final dataset contains *N* = 1290 state-action pairs, with an average of 7.17 pairs per trajectory (range: 1–10), covering 66 fault scenario types. Data quality is ensured through completeness and type-consistency checks for state, action, and reward records, consistency verification between state-action sequence lengths and action legality, and trajectory quality scoring based on restoration rate, power-loss rate, cumulative reward, and action structure. In addition, balanced screening with upper-bound constraints across fault types and trajectory types is adopted to avoid a small number of scenarios or action patterns dominating the dataset. In the final dataset, the expert actions consist primarily of 866 topology-control actions and 422 DER-regulation actions, with the remaining 2 samples corresponding to terminal actions at restoration completion. The retained demonstrations cover diverse fault types, restoration stages, and action structures, including both topology-control and VPP-level DER-regulation actions. Since each demonstration is represented as a graph-structured state-action pair generated from the OpenDSS-based restoration environment, the corresponding operating-state information is embedded in the graph states, which provides feasible and high-quality initialization samples for expert-guided policy learning.

The associated high-level task label $\tau_{i}^{exp}$ is induced from the action semantics by a deterministic and predefined mapping


$$\begin{array}{c} \tau_{i}^{exp}=\phi (a_{i}^{exp}), \end{array}$$ (41)

where ϕ(·) assigns the task category according to the action subset to which $a_{i}^{exp}$ belongs.

During expert-guided pretraining, imitation learning is primarily applied to the high-level task-selection policy. Specifically, the graph-structured state ${𝒢}_{i}$ is encoded using the shared topology-aware encoder $f_{enc}(\cdot )$ described in the state encoding module, yielding a graph-level embedding ${𝐳}_{i}\in {\mathbb{R}}^{d}$. The parameters are optimized by minimizing the cross-entropy loss


$$\begin{array}{c} {ℒ}^{IL}=-{𝔼}_{({𝒢}_{i},a_{i}^{exp})\sim {𝒟}_{exp}}[\log\pi_{\theta }(\tau_{i}^{exp}|{𝐳}_{i})]. \end{array}$$ (42)

The detailed expert-guided pretraining process with topology-aware state encoding is summarized in Algorithm 1. In accordance with PLOS ONE submission guidelines, this algorithm is presented as a non-floating text block.

**Algorithm 1. Expert-Guided Pretraining with GAT-Based State Encoding.**

**Require:** Expert dataset ${𝒟}_{exp}=\{({𝒢}_{i},a_{i}^{exp})\}$, encoder parameters $\theta_{enc}$, high-level policy parameters θ, number of epochs *E*, minibatch size *B*

**Ensure:** Pretrained task-selection policy $\pi_{\theta }(\tau |𝒢)$

1: **for** *epoch* = 1 to *E* **do**

2:  **for** each minibatch $\{({𝒢}_{i},a_{i}^{exp}){\}}_{i=1}^{B}\subset {𝒟}_{exp}$ **do**

3:   Compute embedding ${𝐳}_{i}=f_{enc}({𝒢}_{i};\theta_{enc})$

4:   Infer task label $\tau_{i}^{exp}=\phi (a_{i}^{exp})$

5:   Predict task distribution $\pi_{\theta }(\cdot |{𝐳}_{i})$

6:   Compute loss ${ℒ}^{IL}=-\frac{1}{B}\sum_{i=1}^{B}\log\pi_{\theta }(\tau_{i}^{exp}|{𝐳}_{i})$

7:   Update $\theta_{enc}$ and θ by minimizing ${ℒ}^{IL}$

8:   **end for**

9:  **end for**

10: **return** Pretrained policy $\pi_{\theta }(\tau |𝒢)$

By supervising task-selection behavior through structured action semantics, the IL phase provides a reliable initialization for the high-level policy without requiring additional expert labeling effort. After pretraining, the policy is further refined through reinforcement learning, allowing the agent to adapt beyond the expert demonstrations and optimize long-term restoration performance.

## Case studies and performance analysis

### Experimental setup

This study was conducted on a high-performance computing platform equipped with an AMD Ryzen 7 7800X3D octa-core processor, 32 GB of DDR5 memory, and an NVIDIA GeForce RTX 4060 Ti graphics card, providing sufficient computational power for training and inference of deep reinforcement learning agents.

The test system is a real-world 10 kV distribution network supplied from a 115 kV substation in China, modeled as an 83-node, 97-line graph with two main transformers and dynamic topology reconfiguration capabilities. To emulate a high-penetration active distribution network, Virtual Power Plant (VPP) units are deployed at strategic nodes with specific configurations based on OpenDSS modeling: a 500 kW photovoltaic (PV) station at Node 15; a 500 kW/1000 kWh electrochemical energy storage (ESS) system with 95% round-trip efficiency at Node 45; and an electric vehicle (EV) charging station at Node 23. To reflect demand-side flexibility, the EV station is modeled as a controllable load comprising 300 kW of fixed base load and 200 kW of adjustable charging power, enabling the VPP to modulate power consumption for grid support in unidirectional (grid-to-vehicle) mode.

**Fig 2:**
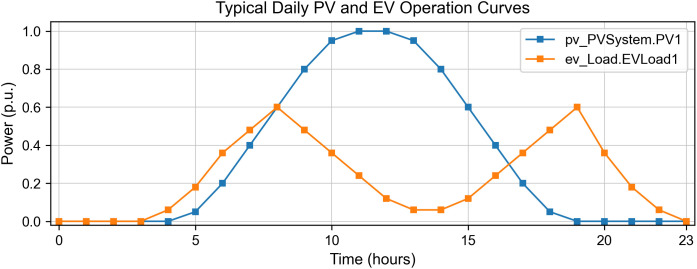
Dynamic test scenario with 24-hour profiles of PV generation and EV charging behavior. The shaded noon interval highlights the risk of reverse power flow and overvoltage.

To capture temporal dynamics beyond static operating conditions, a 24-hour time-series scenario is constructed based on typical daily profiles of PV generation and EV charging behavior (see Fig 2). Notably, the midday period features high PV generation relative to local load, inducing reverse power flow and voltage violations—critical conditions for evaluating the VPP’s auxiliary restoration and voltage support capabilities.

The main training and reward-related hyperparameters are summarized in Table 1. Shared environment and reward settings were kept consistent across all methods, while algorithm-specific optimization parameters followed the configurations listed in Table 1.

All agents shared a common graph encoder for state representation. The encoder consisted of two edge-aware GATConv layers with residual projection, followed by a Transformer encoder and graph-level mean pooling. On top of this shared encoder, DQN used an action-value Q-prediction head, whereas PPO used separate actor and critic heads. Each episode represented a single post-fault restoration trajectory initialized after environment reset with a randomly sampled fault scenario. Episodes were capped at 10 decision steps and could terminate earlier when the terminal action was executed, restoration was successfully completed, or other predefined environment stopping conditions were satisfied.

## Results and analysis

### Comparative evaluation against learning-based and rule-based restoration baselines

To validate the effectiveness of the proposed Expert Knowledge–guided Hierarchical Graph Reinforcement Learning (EH-GRL) method, comparative experiments were conducted against both learning-based and rule-based restoration baselines. For the learning-based comparison, current mainstream deep reinforcement learning methods, PPO and DQN, were first evaluated under identical experimental environments. The reported results of the learning-based baseline comparison among EH-GRL, PPO, and DQN were averaged over three independent training runs with different random initializations.

**Fig 3:**
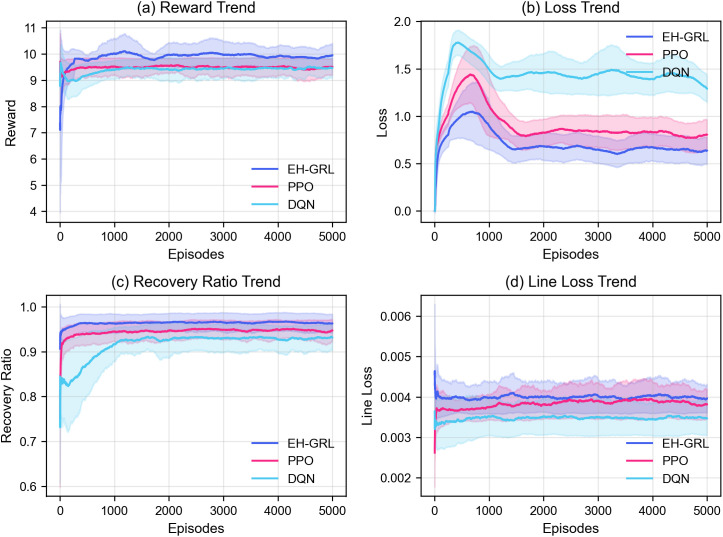
Training performance comparison of EH-GRL, PPO, and DQN algorithms: (a) Reward trend showing faster convergence of the proposed method; (b) Loss trend indicating higher training stability for EH-GRL; (c) Recovery ratio trend demonstrating superior restoration capability of the proposed method; (d) Line loss trend reflecting a slight increase due to higher power recovery.

For fair comparison, EH-GRL and all baseline methods were implemented under the same observation scope, control scope, and candidate action set. Specifically, the heterogeneous control space was unified as a discrete action set consisting of topology-control actions and VPP-oriented DER-regulation actions. For the DER part, each selected action first represents an aggregate VPP-level regulation command, indicating whether the net active-power injection of the corresponding VPP should be increased, maintained, or decreased. This VPP-level command is then realized through the intra-VPP dispatch mechanism described in Section 2.2, where feasible device-level regulation commands and setpoint updates are determined for the controllable PV, ESS, and EV units according to their operating constraints. PPO and DQN operated on the same unified action set, while EH-GRL performed hierarchical task selection followed by task-conditioned action execution. Infeasible topology-control actions and infeasible DER-regulation realizations were filtered using the same legality-check mechanism for all methods, so that the compared approaches differed in decision architecture rather than action availability. The experiments ran for 5000 episodes. Key indicators such as reward, loss function, recovery ratio, and line loss were observed to evaluate the performance of each algorithm in terms of training efficiency and control performance. The overall results are shown in Fig 3.

In terms of convergence during training, the proposed algorithm demonstrates a faster learning speed in the early stages, with the reward curve stabilizing at a high level after approximately 1000 episodes. Based on the statistics of the last 1000 episodes, the average reward reaches 9.8858 ± 0.0301, exceeding those achieved by PPO (9.4679) and DQN (9.4557). The evolution of the loss function further reflects differences in training stability among the three methods: the proposed algorithm attains an average loss of 0.6466 ± 0.0124, which is lower than that of PPO (0.8038) and substantially lower than that of DQN (1.4072). Consistently, the recovery ratio, as the key task execution metric, remains at the highest level throughout training, as shown in Fig 3(c). During the stable phase, the proposed algorithm achieves an average recovery ratio of 96.46% ± 0.0014 with minimal fluctuation, indicating strong robustness, whereas PPO and DQN reach 94.76% and 92.90%, respectively, with DQN exhibiting noticeable instability in the early training stage.

With respect to operational efficiency, Fig 3(d) shows that the average line loss rate of the proposed algorithm is approximately 0.0040, which is slightly higher than that of PPO (0.0039) and DQN (0.0035). This increase is attributable to the algorithm’s strategic preference for restoring a larger amount of load, which shifts the system operating point toward higher power flow levels. Consequently, a moderate increase in line losses is a reasonable trade-off when a substantially higher recovery ratio is achieved.

These results demonstrate that EH-GRL outperforms the learning-based baselines in terms of learning speed, training stability, and restoration effectiveness.

To further examine the influence of the VPP-level DER regulation granularity, a sensitivity analysis was conducted by varying the normalized adjustment ratio δ in the DER action set ${𝒜}_{DER}=\{-\delta ,0,+\delta \}$. The remaining environment settings, reward coefficients, and training settings were kept unchanged. The tested values of δ were set to 0.1, 0.2, and 0.3. For each value of δ, five independent training runs were conducted, and the averaged results were used for plotting and performance evaluation. The evaluation metrics are consistent with those used in the learning-based baseline comparison, including reward, loss function, recovery ratio, and line loss.

**Fig 4:**
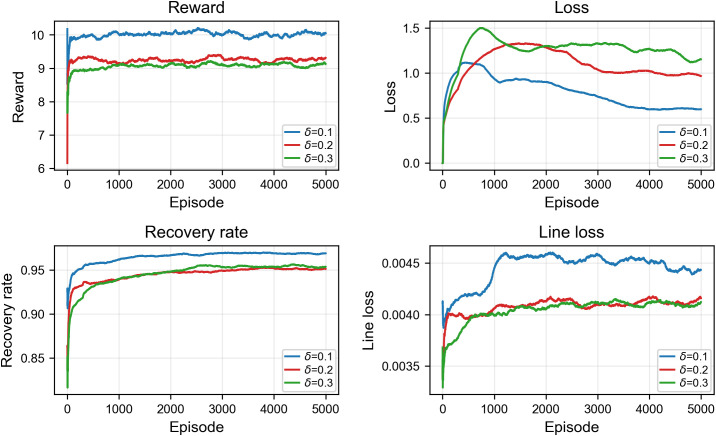
Sensitivity analysis of the VPP regulation step size δ: (a) reward; (b) training loss; (c) recovery ratio; and (d) line loss. All metrics are evaluated under the same training and reward settings, with δ set to 0.1, 0.2, and 0.3.

As shown in Fig 4, δ=0.1 provides the most favorable overall trade-off among the tested settings. Based on the statistics of the last 1000 training episodes, δ=0.1 obtains the highest average reward of 10.0328 and the lowest loss of 0.6018, while achieving a recovery ratio of 96.88%. When δ is increased to 0.2, the average reward decreases to 9.2828, the loss increases to 0.9776, and the recovery ratio decreases to 95.10%. A further increase to δ=0.3 leads to lower reward and higher loss, with an average reward of 9.1030 and a loss of 1.1923, while the recovery ratio remains lower than that obtained with δ=0.1, at 95.42%.

Although the line loss slightly decreases as δ increases, this should be interpreted together with the restored load level. A lower line loss in this case is mainly associated with a lower recovery ratio and reduced power delivery, rather than indicating a better restoration strategy. Overall, a smaller regulation step size provides finer VPP-level DER adjustment and more stable policy learning, whereas a larger δ may lead to coarser DER regulation and less stable training. Therefore, δ=0.1 is adopted in the main experiments as a balanced setting between regulation granularity, restoration effectiveness, and training stability.

To further assess the practical restoration capability of the proposed EH-GRL method, an additional comparison was conducted against a DER-aware branch-exchange heuristic with power-flow screening and post-restoration DER dispatch, denoted as the DER-BE heuristic. Unlike learning-based methods, this heuristic relies on predefined distribution network operation rules and deterministic screening of candidate restoration schemes. For this rule-based comparison, all 80 fault types considered in the test set were evaluated in each repeated run. The reported performance metrics were then obtained by averaging the results over both the fault types and the four repeated evaluation runs, so as to keep the statistical reporting consistent with the learning-based comparisons.

After a fault occurs, the faulted line is first isolated. The DER-BE heuristic then selects tie switches and sectionalizing switches according to branch-exchange rules, so that the restored network topology maintains a radial operating structure. For each candidate topology, OpenDSS power-flow calculation is performed to verify its feasibility. Candidate schemes that fail to converge, form loops, or violate basic operational constraints are discarded. After a feasible topology is obtained, DER dispatch is further considered. Specifically, the controllable DER resources, including PV, ESS, and EV units, are adjusted through candidate output or load-regulation levels. For each candidate DER dispatch scheme, the DER-supported supply capability, unrestored load, line loss, and voltage constraints are evaluated. The final heuristic solution is selected as a feasible restoration scheme that jointly includes switching operations and DER dispatch.

**Fig 5:**
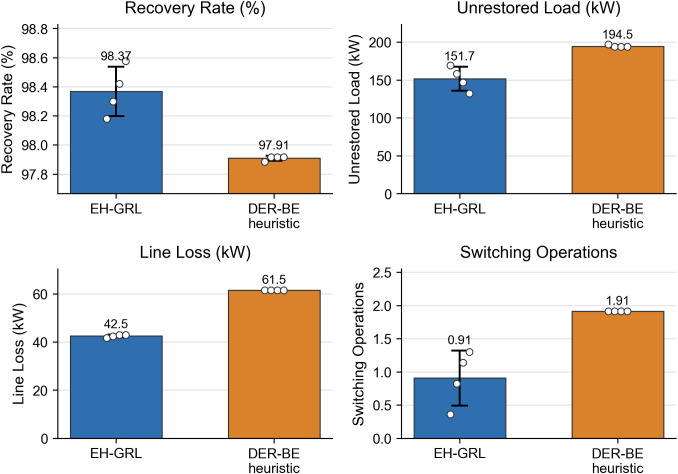
Restoration performance comparison between EH-GRL and the DER-BE heuristic: (a) recovery rate; (b) unrestored load; (c) line loss; and (d) switching operations. DER-BE denotes a DER-aware branch-exchange heuristic with power-flow screening and post-restoration DER dispatch.

As shown in Fig 5, EH-GRL achieves a higher average recovery rate of 98.37%, compared with 97.91% obtained by the DER-BE heuristic. Meanwhile, EH-GRL results in a lower unrestored load of 151.7 kW, whereas the DER-BE heuristic leaves 194.5 kW unrestored. This indicates that the proposed method can identify more effective restoration strategies through hierarchical coordination between topology control and VPP-oriented DER regulation. In terms of network operating efficiency, EH-GRL also achieves a lower line loss of 42.5 kW, compared with 61.5 kW for the DER-BE heuristic. More importantly, the average number of switching operations is significantly reduced from 1.91 for the DER-BE heuristic to 0.91 for EH-GRL. This result suggests that EH-GRL can exploit DER regulation more effectively to support load restoration, thereby reducing the reliance on frequent switching operations while maintaining a high restoration level.

Overall, the comparative results show that EH-GRL not only outperforms learning-based baselines such as PPO and DQN, but also achieves superior restoration performance compared with a DER-aware rule-based branch-exchange heuristic. The results further confirm that the proposed expert-knowledge-guided hierarchical decision framework can improve both restoration effectiveness and operational efficiency.

### Analysis of VPP-assisted support effectiveness

**Fig 6:**
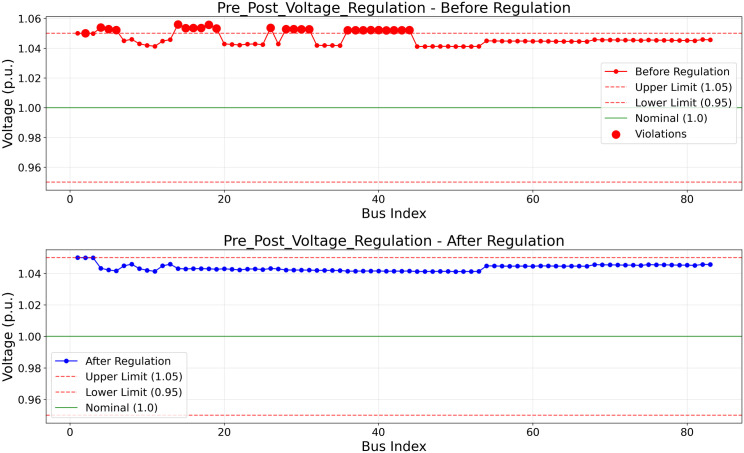
Comparison of bus voltage profiles with and without VPP support. The top panel shows voltage violations caused by PV reverse power flow without VPP participation, while the bottom panel demonstrates effective mitigation of overvoltage and a flattened voltage distribution with VPP-assisted regulation.

As illustrated in Fig 6, the bus voltage profiles with and without VPP participation are compared during peak PV output. This section presents simulation-based validation using the actual distribution system topology. The studied distribution network exhibits a robust structure with a large short-circuit capacity, providing a strong inherent capability to suppress voltage fluctuations.

To investigate the voltage support capability of the VPP under stressed operating conditions, the PV penetration level is temporarily increased by scaling the installed PV capacity to 1 MVA, thereby creating a severe reverse power flow scenario for voltage regulation assessment.

**Voltage regulation performance under high PV penetration.** First, the system operating condition without VPP support is analyzed. As shown in the top panel of Fig 6, when the 1 MVA PV unit operates at full output, limited local load consumption leads to significant reverse power flow. The per-unit voltage at several buses exceeds the upper operational limit of 1.05 p.u., reaching a maximum value of approximately 1.0565 p.u., which results in voltage violations.

Subsequently, the VPP is introduced to participate in grid auxiliary services. As illustrated in the bottom panel of Fig 6, bus voltages are effectively regulated through the coordinated control of heterogeneous DERs. Through this targeted, node-level DER coordination, voltage violations are completely eliminated, voltage peaks are significantly suppressed, and the overall voltage profile becomes smoother and more balanced. These results demonstrate that even in a strong grid environment, VPP-assisted DER coordination plays a critical role in maintaining voltage security.

**Nighttime fault restoration performance without voltage violations.** To further examine whether the benefits extend beyond voltage regulation, additional experiments are conducted under nighttime conditions (21:00), where PV generation drops to zero. In the non-VPP baseline, DER units remain installed in the system but are not aggregated or actively dispatched during restoration; only network reconfiguration actions are allowed. The performance comparison of EH-GRL-based fault restoration strategies is summarized in Table 2.

**Table 2:** Performance comparison of EH-GRL-based fault restoration strategies with and without VPP support under nighttime conditions.

Faulted Line	Method	Restoration Actions	Recovery Ratio	Power Loss (kW)
009041	EH-GRL (with VPP)	Close lines 047048, 010012; ESS discharging at 50% rated; EV load reduction at 20% rated	100%	37.14
	EH-GRL (without VPP)	Close lines 063064, 037038, 043044; Open lines 073075, 069071	97.85%	38.51
014078	EH-GRL (with VPP)	Close line 047048; ESS discharging at 60% rated	100%	37.46
	EH-GRL (without VPP)	Close lines 073074, 079090; Open lines 009047, 069071	97.31%	37.56
005015	EH-GRL (with VPP)	Close line 059060; ESS charging at 50% rated	100%	37.70
	EH-GRL (without VPP)	Close lines 010012, 043044, 063064, 031032; Open line 045051	98.92%	40.98
073075	EH-GRL (with VPP)	ESS discharging at 20% rated	98.92%	38.55
	EH-GRL (without VPP)	Close lines 043044, 063064, 010012; Open lines 045051, 009045	97.31%	36.35
069071	EH-GRL (with VPP)	ESS discharging at 80% rated; EV load reduction at 30% rated	98.92%	38.55
	EH-GRL (without VPP)	Close lines 010012, 063064, 043044; Open lines 008028, 011055	95.70%	35.53

Table notes: ESS indicates energy storage system; EV indicates electric vehicle load.

As shown in Table 2, the VPP-supported EH-GRL strategy achieves better load recovery performance under nighttime conditions. Since PV generation is unavailable at night, the improvement mainly comes from the coordinated use of ESS and flexible EV load. For fault lines 009041, 014078, and 005015, the VPP-assisted strategy restores all interrupted loads, while the non-VPP strategy cannot achieve full restoration. For fault lines 073075 and 069071, although the recovery ratio is still below 100%, it is higher than that of the non-VPP baseline. This indicates that VPP resources can provide additional local support and help cover the remaining load that cannot be restored by network reconfiguration alone.

Compared with the non-VPP strategy, which mainly relies on multiple switching operations to establish alternative supply paths, the VPP-assisted strategy can coordinate ESS discharge, ESS charging, and EV load adjustment to improve restoration flexibility. In terms of power loss, the results should be interpreted together with the restored load level. For example, the VPP-supported strategy reduces the power loss in the case of fault line 005015, while in some other cases the loss slightly increases because more load is restored and more power is delivered through the network. Therefore, the main contribution of VPP support in these nighttime scenarios is reflected in improving load recovery capability and providing additional operational flexibility, while maintaining acceptable operating performance.

### Validation of expert-guided optimization mechanism

This section evaluates the effectiveness of the expert-guided optimization mechanism, with a particular focus on how imitation learning (IL) influences policy decision confidence and training stability. For this purpose, the EH-GRL models used in this subsection are trained as a separate set of experimental instances, with training configurations specifically tailored to the analysis of expert guidance. The evaluation metrics are kept consistent with those adopted across other experimental sections to ensure comparability.

**Fig 7:**
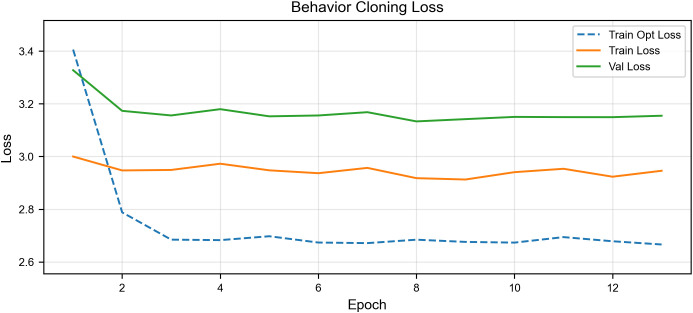
Behavior cloning loss curves during expert-guided imitation learning. The expert demonstration dataset is divided into training and validation subsets with a ratio of 4:1. The dashed curve denotes the total optimization loss used for policy-network updating, while the solid curves represent the behavior cloning losses on the training and validation subsets.

To further examine the training behavior of the imitation learning stage, the expert demonstration dataset was divided into training and validation subsets with a ratio of 4:1. Fig 7 shows the behavior cloning loss curves during expert-guided imitation learning. The dashed curve represents the total optimization loss used for updating the policy network, while the solid curves denote the behavior cloning losses on the training and validation subsets, respectively.

As shown in Fig 7, the total optimization loss decreases rapidly during the first few epochs and then reaches a relatively stable level, indicating that the policy network can effectively learn from the expert demonstrations. The training loss remains lower than the validation loss throughout the imitation learning stage, while both curves exhibit stable trends without severe oscillation. Since the validation loss remains stable and does not show an increasing trend, no obvious overfitting is observed under the adopted 4:1 data split. This result suggests that the expert-guided pretraining process provides a stable supervised initialization for the policy.

**Fig 8:**
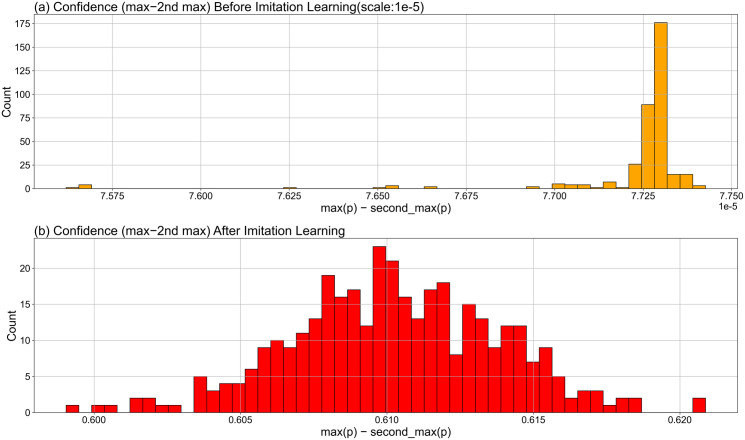
Distribution of action probability gap ΔP before and after imitation learning. (a) Initial random exploration with low decision confidence; (b) After expert-guided imitation learning, showing increased confidence and clearer separation between the most likely and second most likely actions.

After confirming the stable convergence of the imitation learning process, we further analyze its effect on policy decision confidence. Fig 8 illustrates the distribution of the action probability gap ΔP between the most likely action and the second most likely action in the policy output, before and after the imitation learning phase.

As shown in Fig 8(a), before introducing imitation learning (i.e., during the initial random exploration phase), ΔP is extremely small, with its distribution mainly concentrated near 0, corresponding to a magnitude of only 10^−5^. This indicates that without expert guidance, the agent struggles to effectively distinguish between optimal and sub-optimal actions, exhibiting high uncertainty in the decision-making process.

After introducing expert knowledge for imitation learning, the decision characteristics of the policy changed significantly. As shown in Fig 8(b), the distribution of ΔP shifted entirely to the right, centered around 0.610, and presented a concentrated single-peak morphology. Most sample probability differences were concentrated in the 0.600 to 0.620 interval. This result indicates that imitation learning enables the agent to assign higher confidence to actions recommended by experts, thereby forming a clearer and more stable decision preference. This demonstrates that expert knowledge provides effective supervision signals for policy learning, laying a good initialization foundation for the subsequent reinforcement learning phase.

**Fig 9:**
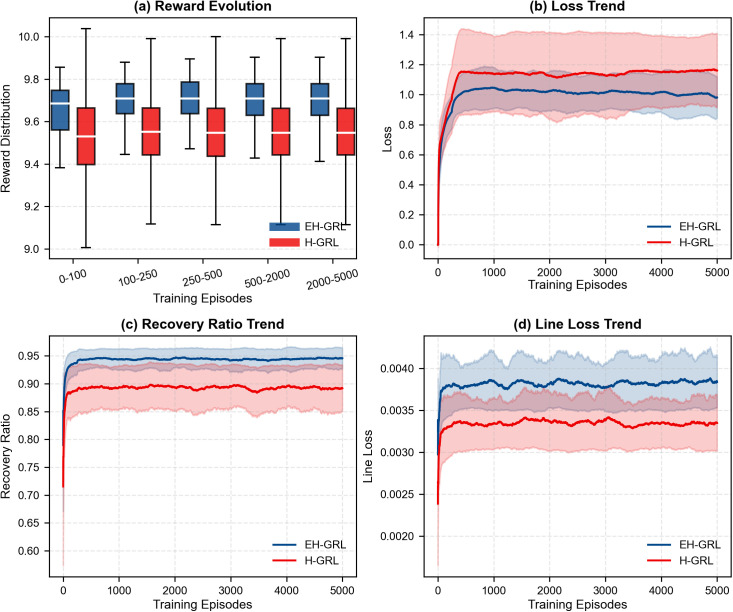
Ablation study comparing EH-GRL and H-GRL methods. (a) Reward distribution showing reduced variance and more consistent performance in EH-GRL; (b) Loss trend indicating smoother convergence and lower estimation bias for EH-GRL; (c) Recovery ratio trend demonstrating higher restoration success rate and policy stability with expert knowledge guidance.

To isolate the contribution of expert knowledge guidance, an ablation study is conducted by removing imitation learning and expert supervision from the proposed EH-GRL, yielding a baseline denoted as Hierarchical Graph Reinforcement Learning (H-GRL). The reported results of this ablation comparison were averaged over three independent training runs with different random initializations. Fig 9 compares the two methods in terms of training stability and fault restoration performance. EH-GRL demonstrates faster convergence and superior stability in the core restoration metric. As shown in Fig 9(c), EH-GRL achieves an average recovery ratio of 94.51%, whereas H-GRL attains 89.26%, with the standard deviation reduced from 0.17% to 0.08%. Consistent improvements are also observed in the loss function (Fig 9(b)), where EH-GRL converges to a lower and smoother level (1.0023 ± 0.0087), while the corresponding value for H-GRL is 1.1572 ± 0.0051, indicating more accurate value estimation and enhanced training stability.

Regarding the reward metric (Fig 9(a)), H-GRL attains a slightly higher average reward during the stable period, reaching 9.5820, while EH-GRL yields an average reward of 9.5117. However, the reward variance of H-GRL is nearly twice that of EH-GRL, amounting to 1.1966 and 0.6095, respectively. This observation suggests that although the removal of expert guidance may encourage more aggressive exploration, it also introduces substantially higher uncertainty and operational risk. In contrast, the more concentrated reward distribution of EH-GRL reflects safer and more reliable decision-making, which is more desirable for safety-critical fault restoration scenarios.

Synthesizing the above results, for power system restoration scenarios with extremely high requirements for safety and robustness, EH-GRL incorporates safety constraints derived from expert knowledge into the policy learning process. While making a moderate trade-off in average economic indicators, it achieves an approximately 5.25% increase in restoration success rate and significantly reduces the volatility of policy output. Although EH-GRL exhibits a slightly higher line loss compared with H-GRL, which achieves 0.0033 ± 0.0006, its overall performance demonstrates clear advantages in terms of safety and stability. These results demonstrate that the proposed Expert-Guided Optimization learning mechanism better meets the requirements for safe and stable system operation in practical engineering applications.

## Conclusion

This paper presents a hierarchical graph reinforcement learning framework for fault restoration in high-penetration active distribution networks. By incorporating VPP–based aggregation control and expert-guided optimization collaboration, the proposed method enables efficient and scalable decision-making for complex source–grid–load systems. The hierarchical design, featuring GNN-based state representation and a high-level mode selection–low-level action execution mechanism, effectively addresses heterogeneous action spaces and alleviates the training instability commonly encountered in large-scale power grid reinforcement learning problems. Simulation results demonstrate that the proposed approach significantly improves load recovery performance while reducing voltage violation risks under high photovoltaic penetration, outperforming traditional topology reconfiguration strategies and mainstream deep reinforcement learning methods in terms of robustness and policy stability.

Future work will focus on enhancing the robustness of the proposed framework under non-ideal operating conditions, including communication delays and data packet loss. In addition, the collaborative optimization and game-theoretic interactions among multiple VPPs in market-oriented environments will be investigated to further support the development of resilient, flexible, and intelligent distribution networks.
